# Neoadjuvant/Perioperative Treatment Affects Spatial Distribution and Densities of Tumor Associated Neutrophils and CD8+ Lymphocytes in Gastric Cancer

**DOI:** 10.3390/jpm11111184

**Published:** 2021-11-12

**Authors:** Anne Hoffmann, Hans-Michael Behrens, Steffen Heckl, Sandra Krüger, Thomas Becker, Christoph Röcken

**Affiliations:** 1 Department of Pathology, University Hospital Schleswig-Holstein, Campus Kiel, D-24105 Kiel, Germany; anne.hoffmann96@web.de (A.H.); behrensm@path.uni-kiel.de (H.-M.B.); Sandra.Krueger@uksh.de (S.K.); 2 Department of Internal Medicine II, University Hospital Schleswig-Holstein, Campus Kiel, D-24105 Kiel, Germany; steffen.heckl@uksh.de; 3 Department of General Surgery, Visceral, Thoracic, Transplantation and Pediatric Surgery, University Hospital Schleswig-Holstein, Campus Kiel, D-24105 Kiel, Germany; thomas.becker@uksh.de

**Keywords:** gastric cancer, tumor associated neutrophils, cytotoxic T cells

## Abstract

Tumor associated neutrophils (TANs) and cytotoxic T cells (CTLs) are part of the tumor microenvironment of gastric cancer (GC). We explored their tumor biological significance in neoadjuvantly/perioperatively treated GC. Immunostaining was performed on whole tissue sections of 173 GCs, using antibodies directed against myeloperoxidase (MPO) and CD8. Stained specimens were digitalized, and the densities of TANs and CTLs were assessed separately in the mucosa, tumor surface, tumor center, invasion front, and tumor scar. The densities were correlated with clinicopathological patient characteristics. Compared with a historical cohort of 449 treatment naive GCs, the TAN density in the invasion front was significantly lower in neoadjuvantly/perioperatively treated GCs. TAN density in the tumor center and invasion front correlated with tumor regression. TAN density also correlated with CTL density in the tumor center and invasion front. A high density of CTL in the tumor center correlated with an improved overall survival and tumor specific survival. We show that neoadjuvant/perioperative (radio-) chemotherapy impacts on the immune microenvironment of GC, while also depending on sex. The density of TANs in neoadjuvantly/perioperatively treated GCs differed from findings made in a treatment naive GC cohort.

## 1. Introduction

Gastric cancer (GC) is the fifth most common cancer in the world. Due to late diagnosis, patient prognosis is often dismal. Moreover, its incidence shows substantial geographic variability, for example, being high in East Asia and low in Western countries [1]. However, the overall incidence of cancer of the distal stomach is decreasing, while it is steadily increasing for adenocarcinomas of the cardia and gastroesophageal junction. The etiology of both is diverse. Known risk factors include an infection with Helicobacter pylori, smoking, higher age, high salt intake, obesity, chronic gastroesophageal reflux disease, and low physical activity [2,3]. Interestingly, the risk of suffering from GC is gender specific [4]. Men are two times more likely to develop a distal tumor and five times more likely to develop a proximal GC compared with women. However, not only is the development of cancer increased for men, also the mortality for men from the cancer is twice as high as for women [5]. One putative explanation for this gender-specific difference could be the sexual dimorphism of the immune system [4,6] and lifestyle differences [7]. The most important treatment for GC is surgery, while neoadjuvant (radio-) and/or perioperative chemotherapy are administered in a more locally advanced tumor stage [8]. In the palliative setting, additionally biologicals have become the standard of care, such as trastuzumab and ramucirumab [9,10]. More recently, immune-checkpoint inhibitors have gained considerable attention and are novel treatment options in the adjuvant [11] and palliative settings [12,13]. This also brought the tumor immune microenvironment (TIME) into focus, as it may affect patient outcomes and treatment efficacy.

The TIME can be categorized into three different groups: (a) the infiltrated-excluded TIME, which is characterized by the exclusion of cytotoxic T cells (CTL) from the tumor core; (b) the infiltrated-inflamed TIME, which is observed in immunologically ‘hot’ tumors and characterized by a high level of infiltration of CTLs expressing PD-1, and leukocytes and tumor cells expressing the immune-dampening PD-1 ligand PD-L1; and (c) a subclass of infiltrated-inflamed TIME, i.e., TLS-TIME, which includes tertiary lymphoid structures and lymphoid aggregates, whose cellular composition is similar to that found in lymph nodes [14]. Both, tumor genotype/phenotype and immunological composition, are a function of TIME. Interestingly, retrospective studies of different patient populations treated with immune-checkpoint inhibitors demonstrated TIME-dependent treatment efficacy [15,16]. Major cellular components of TIME are tumor-associated neutrophils (TANs) and CD8+ T cells (CTLs). TANs play an important role in the tumor microenvironment and progression [17,18]. TANs are often correlated with a lower survival rate for different tumor types and have been used as a prognostic biomarker in comparison with lymphocyte count [19]. However, TANs can also have a positive effect on survival. We, and others, have shown a gender specific effect for GC [20,21,22]. For women with GC, the TAN density, especially located in the invasion front, was an independent predictor of tumor-specific survival; in contrast to men, where no association was found [20,21].

In addition, CTLs play an important role in TIME. A link has been described between Lauren classification, CTL-densities, and a positive prognostic outcome [23,24]. Furthermore, adult women showed higher CD4/CD8 ratios, elevated CD4+ T lymphocytes, increased T cell activation and proliferation, and lower CD8+, Treg, and NK cells. This could be an explanation for the stronger innate and adaptive immune responses in women, reducing the overall mortality from cancer [25,26]. Furthermore, CD8+ cells can also be used to predict the success rate of immunotherapy [27] and, hence, patient outcome [28].

Leukopenia and neutropenia are well-known systemic side effects of neoadjuvant/perioperative therapy in GC. In the MAGIC trial, approximately 11.1% of the patients sustained postoperatively from grade 3–4 leukopenia, while grad 0, 1, or 2 leukopenia were observed in 88.9% of the patients. In the CROSS study, any-grade leukopenia was found in 60% of the patients and neutropenia in 9%. Evidence is increasing that neoadjuvant/perioperative therapy also affects the TIME and its cellular composition. Based on our findings made in treatment naïve GCs, we here aimed to test the hypothesis that neoadjuvant/perioperative treatment affects TAN densities and CTL counts locally in GC. We also specifically addressed putative gender specific differences, such as gender specific responses to chemotherapy and differences in the immune infiltrate.

## 2. Materials and Methods

### 2.1. Ethics

All executed procedures were in accordance with the ethical standards of the responsible committee on human experimentation (institutional and national) and with the Helsinki Declaration of 1964 and later versions. Ethical approval was obtained from the local ethical review board (D 453/10). All experimental work complied with all mandatory laboratory health and safety procedures.

### 2.2. Study Population

In the study population, all patients who had undergone a total or partial gastrectomy for adenocarcinoma of the stomach or gastroesophageal junction and who were treated with a neoadjuvant or perioperative (radio-) chemotherapy between 1998 and 2019 (hereinafter referred as ‘neoadjuvant GC’) were included. Tissue specimens were collected from the archive of the Department of Pathology, University Hospital Schleswig-Holstein, Campus Kiel. Information regarding tumor location, age at diagnosis, gender, tumor type, tumor grade, residual tumor status, tumor size, depth of invasion, number of lymph nodes resected, and number of lymph nodes with metastases was sought from the electronic database of the Department of Pathology. Inclusion criteria were a histologically confirmed primary adenocarcinoma of the stomach or gastroesophageal junction and having received chemotherapy (Table S1). Exclusion criteria were defined as a histology identified tumor type other than adenocarcinoma or patients who were not treated with neoadjuvant/perioperative (radio-) chemotherapy. For comparison, we used a treatment naïve cohort who had undergone a total or partial gastrectomy between 1997 and 2009 without neoadjuvant therapy (Table 1) [21]. From the Epidemiological Cancer Registry of the state of Schleswig-Holstein, Germany, was obtained the date of the patient death. From hospital records and general practitioner follow-up, information of patients who were still alive was retrieved. After inclusion in the study, all related patient data were pseudonymized.

### 2.3. Histology

Specimens were fixed in formalin and embedded in paraffin (FFPE). Paraffin sections were stained with hematoxylin and eosin. Tumors were classified according to Laurén. The pTNM-stage of all study patients was defined, according to the eighth edition of the International Union Against Cancer guidelines [29]. We also included lymphatic invasion (L-category), venous invasion (V-category), and residual tumor classification (R-category) [29]. Tumor regression was categorized according to Becker et al. [30] into tumor regression grade (TRG) 1a (complete regression), TRG 1b (<10% vital tumor cells), TRG2 (10% to 50% vital tumor cells), and TRG3 (>50% vital tumor cells).

### 2.4. Myeloperoxidase and CD8+ Immunohistochemistry

For immunostaining, we used the Bondmax automated slide staining system (Leica Microsystems, Wetzlar, Germany), a rabbit polyclonal anti-human-MPO antibody (dilution 1:2000; Dako, Carpinteria, CA, USA), and a monoclonal mouse anti-human CD8 antibody (1:100; Dako, Carpinteria, CA, USA) diluted with Bond Primary Antibody Diluent (Leica, Newcastle, UK). Immunostaining was visualized with a Bond Polymer Refine Detection Kit (Leica Biosystems, Newcastle, UK). Bond Epitope Retrieval Solution 2 (Leica Biosystems, Newcastle, UK) was used to carry out automated antigen retrieval at 20 min. The omission of the primary antibody served as negative control.

### 2.5. Image Analysis and Virtual Microscopy

Immunohistochemically stained tissue sections were digitalized using a Leica SCN400 microscopic whole-slide scanner (Leica Biosystems, Nussloch, Germany) at its maximum, nominally 40-times, magnification. The pixel-to-pixel distance equaled 0.25 µm in the virtual image. The scanning images were exported from the scanner system as Leica SCN format files. To detect MPO^+^ and CD8^+^ cells semi-automatic image analysis with Definiens Tissue Studio (version 3.6.1, Definiens, München, Germany) was performed at 20-times magnification. Software settings were used to vary the programmed outputs and classify the desired cells. Tissue background separation distinguished between tissue and the background (auto threshold; multiple tissue pieces; 10,000 µm^2^ minimum tissue size). Nuclei were detected (nucleus detection: 0.1 hematoxylin threshold; 50 µm^2^ typical nucleus size) and virtual cell borders were designed (cell simulation: simulation mode = grow from nuclei; 1 µm maximum cell growth). Depending on the intensity of brown chromogen (general settings: stain combination = IHC brown chromogen; IHC marker = cytoplasm) the designed cell was classified as TAN (cell classification: selected feature = IHC marker intensity; measurement in = cell; threshold none/low = 0.5). Analyses were exported as CSV files, which again had to be converted in order to be used. The viewer and painting program VMP was used to mark five tumor compartments (see below).

### 2.6. Marking Compartments

Five, non-overlapping compartments of the tumor were discriminated manually with the VMP painting program: non-neoplastic peritumoral mucosa, tumor surface, tumor center/tumor scar, and invasion front. Marking the peritumoral mucosa, a whole tumor-free area between muscularis mucosa and the mucin layer was selected. For the compartment tumor surface, we marked an area from the surface up to 500 μm into the tumor, avoiding the necrotic layers directly covering the luminal tumor surface. For the tumor center, most parts of the tumor were captured and neutrophil abscesses were excluded. The invasion front was marked up to a width of 250 μm and could include small parts of the surrounding stroma. As tumor scar, we marked the tissue area that had responded to neoadjuvant therapy without residual tumor cells. Tissue free space (e.g., artifacts generated during the cutting of the FFPE tissue samples), large necrotic areas, or neoplastic glands wider than 200 μm filled with neutrophil debris and apoptotic bodies were avoided.

The TIME classes were assessed, as described by Binnewies et al. [14], and using CD8-immunostained tissue sections. In brief, we selected the infiltrated-excluded GC samples surrounded by CTLs. The infiltrated-inflamed type was characterized by CTLs in the tumor center, while TLS–TIME also enclosed tertiary lymphocyte structures, which were similar to lymph nodes (see below).

### 2.7. Study Design

**Figure 1:**
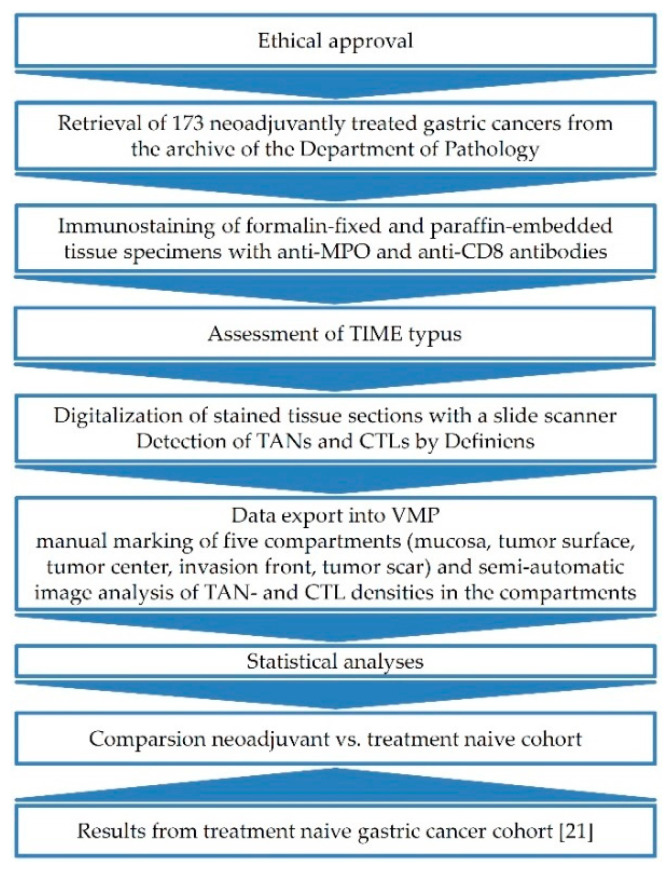
Schematic overview of the study methodology.

Whole tissue sections from neoadjuvant GCs were stained with an antibody directed against MPO or CD8. The different densities of MPO^+^ TAN and CD8^+^ CTL were computed for the tumor compartments, such as the tumor surface, tumor center/tumor scar, invasion front, and peritumoral area. The counts were correlated with clinicopathological patient characteristics and survival. Furthermore, we included data obtained from our former treatment naïve cohort to compare our outcome with the previous results [21]. Figure 1 provides a schematic overview of the study design.

### 2.8. Statistical Analysis

For statistical analyses we used SPSS 24.0 and 25.0 (IBM Corporation, New York, NY, USA). First, raw score values from the TAN and CTL densities were dichotomized at the median and, regarding TAN densities, also divided into four groups, by splitting into quartiles (Q1, Q2, Q3, and Q4). Subsequently, quartiles were grouped into a TAN-low (Q1) and a TAN-high (Q2, Q3, Q4) group (according to our treatment naïve cohort) [21], and into CTL-low (Q1, Q2) and CTL-high (Q3, Q4). A Fisher’s exact test was used for testing the significance of correlation between clinicopathological variables. In contrast, Kendall’s tau test was instead used for ordinal scale values for calculation. Overall (OS) and tumor specific survival (TSS) was computed using the Kaplan-Meier method and compared by log-rank test to determine the significance of differences between the survival curves. OS and TSS were defined as time from surgery to patient death. The cohort was also separated by gender and raw score values, OS and TSS were calculated again. For the correlation between TANs and CTLs, we used Pearson’s correlation and scatter plot graphs. To estimate their impact, clinicopathological features were correlated by gender and their effect on survival. In addition, a multivariate Cox regression model was carried out, counting in all factors with *p* ≤ 0.10 at univariate analysis. To compensate for the false discovery rate within the correlations, we applied the Simes (Benjamini-Hochberg) procedure (false discovery rate (FDR)-correction) [31]. A *p*-value ≤0.05 was considered statistically significant.

## 3. Results

In total, 173 patients could be included in the study (Table 1). At the time of diagnosis, the median age was 65.2 years (range years 20.8–81.7 years) and 134 (77.5%) patients were male and 39 (22.5%) female. While, 79 (45.7%) GCs had an intestinal phenotype according to Lauren, 30 (17.3%) had a diffuse phenotype, 33 (19.1%) were mixed, and 18 (10.4%) were unclassifiable. Overall survival was available in 154 cases.

### 3.1. Density of TAN and CTL as a Function of the Tissue Compartment

**Figure 2:**
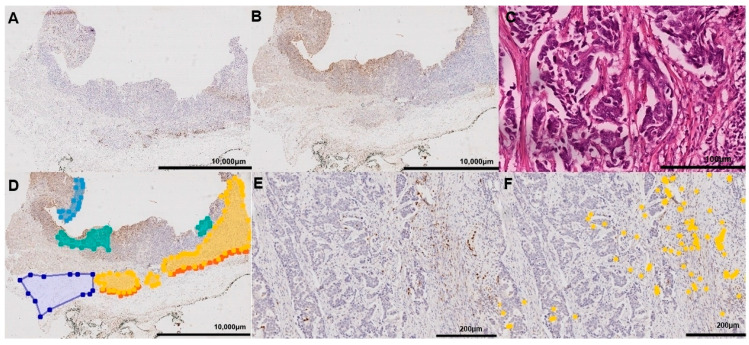
Digital image analysis. Digital image analysis was used to quantify the spatial distribution of tumor-associated neutrophils (TANs; **A**,**D**,**E**,**F**) and cytotoxic T cells (**B**) in neoadjuvantly treated gastric cancer. The viewer and painting program VMP was used to mark the tumor compartments (**D**): mucosa (light blue), tumor surface (green), tumor center (yellow), invasion front (orange), and tumor scar (dark blue). The density was quantified by image analysis using Definiens Tissue Studio^®^ (TANs identified by Definiens are marked as yellow points; (**F**). The same tumor is pictured in all figures. Anti-myeloperoxidase immunostaining (**A**,**D**,**E**,**F**); anti-CD8 immunostaining (**B**); hematoyxlin and eosin-staining (**C**).

First, we investigated the distribution of TAN and CTL in five different compartments; i.e., the peritumoral non-neoplastic mucosa, tumor surface, tumor center, invasion front, and tumor scar (Figure 2). Tumor center and tumor scar differed from each other by the presence or absence of residual tumor cells, e.g., due to complete regression (TRG1a) according to Becker et al. [30]. As shown in Table 2, the median density (n/mm^2^) of TAN differed significantly between the five different compartments (*p* < 0.001). At the tumor surface was found the highest median density (486.6 TAN/mm^2^), with the lowest in the tumor scar (36.8 TAN/mm^2^). With regard to CTL, densities also varied significantly, being the highest at the invasion front (420.7 CTL/mm^2^) and the lowest in the tumor scar (79.5 CTL/mm^2^) (*p* < 0.001).

### 3.2. Correlation between TAN and CTL Densities

We then correlated TAN density with CTL density in five different compartments (Table 2). In all compartments, the median density of CTLs was higher than the median density of TANs, except for the tumor surface, where the density of TANs was higher than the density of CTLs. The largest difference was found in the invasion front (TAN vs. CTL, 134.8/mm^2^ vs. 420.7/mm^2^). In addition, comparing TAN and CTL density for each compartment, the densities of TAN and CTL correlated significantly with each other in the tumor center (*p* = 0.001), invasion front (*p* = 0.002), and tumor scar (*p* = 0.027, data not shown). CTL were not available from the treatment naïve cohort.

### 3.3. TIME-Classes in Neoadjuvantly Treated GC

**Figure 3:**
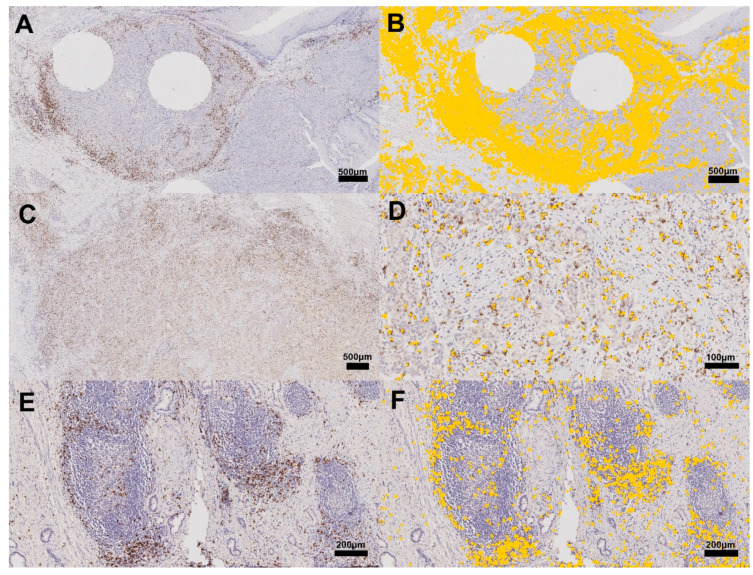
General types of TIME. We used the definition of the different types of TIME by Binnewies et al. [14] to divide our cohort. Samples were stained with an anti-CD8+ antibody (**A**–**F**). The cytotoxic T cells (CTLs) were marked with a yellow dot using the Definiens Tissue Studio (**B**,**D**,**F**). In the infiltrated-excluded phenotype CTLs are around the tumor (**A**,**B**). In the infiltrated-inflamed phenotype CTLs are in the tumor center (**C**,**D**). In the TLS-TIME phenotype tertiary lymphoid structures and lymphoid aggregates are found (**E**,**F**).

TIME [14] was assessable in 133 (76.9%) cases (Figure 3). In 40 cases (23.1%) TIME could not be assessed, either due to marked (27 (15.5%) cases) or complete tumor regression (13 (7.5%)), prohibiting a valid assessment of the TIME type. Finally, 30 (22.6%) of 133 assessable tumors were classified as infiltrated-excluded, 75 (56.4%) as infiltrated-inflamed, and 28 (21.1%) as TLS-TIME. The three TIME classes correlated with CTL density at the invasion front (*p* < 0.001). Interestingly, TIME classes were also associated with tumor regression; 83% of the GCs with an infiltrated-excluded TIME showed no tumor regression (TRG3) compared with 36% of the TLS-TIME. Interestingly, the TIME classes were also associated with UICC stage and ypL-category. No association was found with, e.g., tumor type, ypT-category, or patient survival (Table 3).

### 3.4. Correlation of the Expression of TANs or CTLs with Clinicopathological Patient Characteristics

Next, we correlated the densities of TANs and CTLs in GC with different clinicopathological patient characteristics. According to Clausen et al. [21] TAN density was dichotomized into TAN low (Q1) and TAN high (Q2-Q4) using quartile ranges (Table S2). Following this dichotomization, TAN density at the invasion front was associated with the ypT-category (TAN high was more common in higher ypT-stage), in the tumor scar with anatomical localization (TAN high was more common in proximal GCs), and in the tumor center, as well as at the invasion front, with tumor regression (TAN high was more common in tumors with little or no regression) (Table S2; not significant after correction for multiple testing).

CTL density was dichotomized at the median into CTL low and CTL high (Table S3). Following this dichotomization, CTL density in the tumor center was associated with ypN-category (CTL high was more common in low ypN-stages) and UICC stage (CTL high was least common in stage IV tumors), and in the invasion front with perineural invasion and tumor regression (CTL high was more common in tumors with perineural invasion and without regression; Table S3). After correction for multiple testing, the correlation between CTL density at the invasion front and tumor regression remained significant (*p* < 0.001; Table S3).

### 3.5. Prognostic Significance of TANs and CTLs 

**Figure 4:**
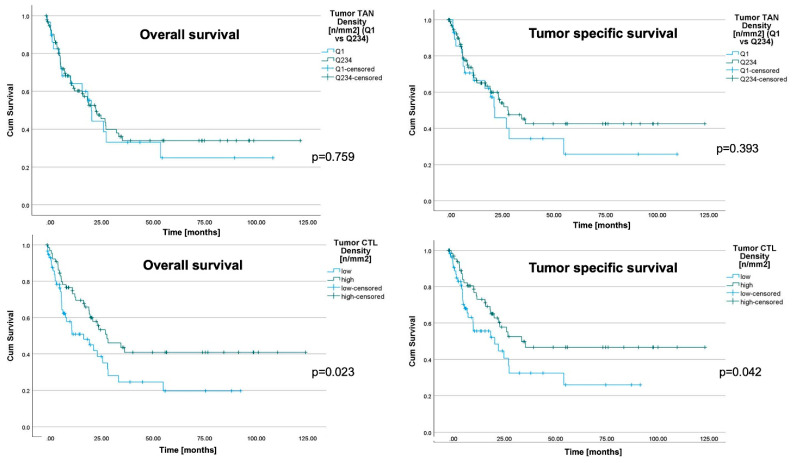
Patient survival. Kaplan-Meier curves of the whole cohort, depicting patients’ overall and tumor-specific survival, according to the densities of myeloperoxidase-immunoreactive tumor-associated neutrophils (TAN) in tumor center (dichotomized into quartile 1 vs. quartile 2–4) and densities of CD8+ cells (dichotomized into quartile 1 and 2 vs. quartile 3 and 4). All *p*-values shown in the graph were obtained by log-rank test. Patients with a higher cytotoxic T cell (CTL) density in the tumor center showed a significantly better overall and tumor specific survival. In contrast, the TAN density in the tumor center was not correlated with a better outcome.

We then compared the dichotomized densities of TAN (Q1 vs. Q2–4) and CTL (Q1/Q2 vs. Q3/4) with patient survival (Figure 4). While no significant difference was found between TAN and OS or TSS (Table S2). The OS and TSS showed a difference of CTLs in the tumor center. Patients with more than 296.2 CTLs/mm^2^ in the tumor center showed a median OS and TSS of 31.7 and 39.0 months, respectively, compared with 19.3 and 24.6 months in the CTL low group, respectively (Table S3).

### 3.6. Comparison of TAN Densities in Neoadjuvantly Treated with Treatment Naïve Gastric Carcinomas

We next compared the density of TANs in neoadjuvantly treated GC with our previously published data on treatment naïve GCs [21]. In four compartments, i.e., mucosa (*p* < 0.001), tumor surface (*p* = 0.006), tumor center (*p* = 0.513), and invasion front (*p* = 0.004) (data not shown), the median density of TANs was always lower in neoadjuvantly treated GCs compared with treatment naïve GCs, except for the mucosa (Table 2).

### 3.7. Impact of Gender on TAN and CTL Densities

Previously, we found gender specific differences regarding the biological significance of TANs: TAN density in the invasion front was an independent predictor of TSS only for females [21]. Finally, we explored the impact of gender. No significant difference was found between men and women regarding TAN and CTL densities, respectively (Table 4).

However, when we split the cohort according to gender, TAN densities in the tumor center dichotomized into low (Q1) and high (Q2–4) correlated with tumor regression only in men (*p* = 0.008, Table 4). TAN density at the invasion front only correlated with tumor regression in women (*p* = 0.015) lending further support to the hypothesis that men and women show subtle gender specific differences in their immune responses. No further differences were found between men and women (data not shown).

## 4. Discussion

In this study, we investigated the densities of TANs and CTLs in the tumor microenvironment of neoadjuvantly treated GCs. To support previous results regarding TANs in a treatment naïve GC published in 2020 [21], and to assess the local effect of neoadjuvant/perioperative treatment on TIME in GC, we had specifically chosen the latter cohort.

Our current study is not a follow-up of the original, former patients, who fall in to the ‘pre-MAGIC era, before neoadjuvant/perioperative treatment had become the standard of care. However, the same antibodies and staining procedures were used, and we applied similar evaluation criteria and examined patients from the same Central European catchment area. This similarity of geographic accrual was used as a basis to compare TAN densities with our previous study, in an effort to allow for an approximation of the impact chemotherapy has on TIME. The different cohorts share similarities and differences, which reflect the epidemiological developments of recent years. Both cohorts show a male preponderance and are similar with regard to median patient age and the intestinal phenotype. Nonetheless, the number of proximal tumors was twice as high in the neoadjuvantly treated cohort (69.4%) compared with the treatment naïve cohort (31.8%). There was a difference in cohort size, with 449 treatment naïve cases versus 173 neoadjuvantly treated cases. The absolute number of proximal GCs is representative in both cohorts (143 vs. 120). We found a big difference in distal cases (297 vs. 53). This suggests that patients with distal tumors were more likely to undergo primary surgery than neoadjuvant chemotherapy. However, for tumor progression, the percentages of the different pT-categories differed, with ypT4 accounting for 6.4% in the neoadjuvantly treated cohort and 35.4% in the treatment naïve cohort. Minor differences were also found in ypT1 vs. pT1 (13.9% vs. 11.6%), ypT2 vs. pT2 (13.3% vs. 11.8%), and ypT3 vs. pT3 (56.6 vs. 41.2%), and this may be related to therapy-induced down-staging. Thus, while the comparison of the neoadjuvantly treated cohort with a ‘historical’ treatment naïve cohort of GCs has limitations, it still may provide valuable clues about the impact neoadjuvant treatment has on TIME.

### 4.1. Neoadjuvant Therapy Significantly Reduces TAN Density in Tumor Tissue

Leukopenia and neutropenia are well-known side effects of neoadjuvant therapy. In the MAGIC trial, approximately 11.1% of the patients sustained grade 3–4 leukopenia postoperatively, while grad 0, 1, or 2 leukopenia were observed in 88.9% of the patients [32]. In the CROSS study, any-grade leukopenia was found in 60% of the patients and neutropenia in 9% [33]. Our results support the contention that this well-known systemic effect of neoadjuvant therapy may also lead to a significant overall local reduction of TAN densities within the primary tumor (Table 2). In this respect, it is interesting to note that the significant differences of TAN densities within the different compartments, i.e., tumor surface vs. tumor center vs. invasion front, were maintained (Table 2). Only the mucosa had higher TAN densities in the neoadjuvantly treated GCs compared with the therapy-naïve cohort, for which we have no explanation. Since we did not assess CTLs in the treatment naïve cohort, currently no comment can be made regarding the effect of neoadjuvant treatment on CTLs in our catchment area. However, others have shown that a higher density of tumor infiltrating lymphocytes (TIL) in GC might be associated with a superior OS, a lower depth of invasion, a lower lymph node infiltration, and a lower TNM and UICC stage [34]. Our results showed similar correlations in the tumor center for the OS and pN-category, which underscores the significance of TILs in GC even after neoadjuvant therapy. Besides, a high TIL count might improve the outcome of patients treated with FOLFOX and, thus, TILs may have an effect on the efficacy of chemotherapy [23]. Our data lend further support to these data; CTL counts in the tumor center were associated with a better patient outcome (Table S3).

### 4.2. Therapeutic Response Is Linked to Changes in the Tumor Immune Microenvironment

Apart from the systemic, i.e., more general, immunosuppressive effect neoadjuvant treatment has on cell counts of inflammatory cells, we also observed local effects: tumor regression correlated with TIME, as well as TAN and CTL densities. TAN and CTL counts decreased with increasing therapeutic efficacy in the invasion front (TAN and CTL) and in the tumor center (TAN, Table S2). Concordantly, the number of GCs with an infiltrated-excluded and a TLS-TIME declined with increased efficacy while the percentage of GCs with an infiltrated-inflamed TIME increased (Table 3). Collectively these data show that neoadjuvant treatment has a systemic and a local effect on TIME, which affects both cellular components and TIME groups. These findings are in line with observations made by our group regarding the immune checkpoint molecules PD-L1 and VISTA [35], which showed distinct changes in their expression patterns. The percentage of VISTA-positive cases increased from 8.8% in treatment naïve GC to 30.9% in neoadjuvantly treated GC, while the percentage of PD-L1 positive cases decreased from 23.9% to 15.1% [35].

However, the TIME is suggested to play a key role in tumor biology [14]. The number of TANs, NK-cells, and CTLs change after chemotherapy [36]. In addition, the individual patient response to therapy is highly variable, as was also shown in our cohorts with different numbers of TANs and CTLs in the compartments. Reasons for this might be sex, the patient’s individual immune system, and genetic factors [4,37].

Currently, chemotherapy is the backbone of the neoadjuvant/perioperative treatment of GC. More targeted approaches use monoclonal antibodies directed against the human epidermal growth factor receptor 2 (HER2) or the vascular endothelial growth factor receptor (VEGFR) in the second line palliative regimen [9,10]. However, immune-checkpoint inhibitors are increasingly being explored in diverse tumor types, including advanced GC [38]. Currently, it is difficult to give every GC patient a personalized therapy, and in most of the cases we follow guidelines and apply basic treatment regimens, such as the MAGIC or FLOT protocols [8]. However, recently, the addition of immune-checkpoint inhibitors to the adjuvant regimen improved disease-free survival by 11.4 months in resected esophageal and gastroesophageal junction cancer [11]. Besides, the response to therapy is not uniform, and the exploitation of predictive biomarkers, such as PD-L1, TILs, and TIME, is urgently needed. Patients with a higher intratumoral CD8+ density are associated with a better outcome and also a better therapy response [34]. Likewise, we showed here that CTLs in the tumor center correlated with ypN-category, UICC stage overall, and tumor specific survival. Furthermore, therapy response had an impact on the cell counts of both TANs and CTLs. Thus, future studies should further explore the utility of TANs and CTLs in the prediction efficacy of both neoadjuvant treatments, as well as immune-checkpoint inhibitors.

### 4.3. Sex and Its Impact on the Immune Response after Chemotherapy

Sex influences the development and progression of cancer [39]. Specifically, the immune response of men and women is different [26,40,41]. Furthermore, the therapy response after an immune-checkpoint inhibitor therapy is for women less effective than for men [42]. One reason for this is the higher antigenicity in male cancers. In contrast, the combination of chemotherapy and immune-checkpoint inhibitors is more effective for women [4]. The differences in innate and adaptive immune system and hormones most likely influence response rates [7,26]. In our study we showed that there was no significant difference between men and women for TAN or CTL densities, respectively, in every compartment. However, we found a gender specific tumor regression at the tumor center and at the invasion front (Table 4). Interestingly, we found no effect on the OS, including gender and TANs, in contrast to the chemotherapy naïve cohort [21]. Moreover, patient numbers were low, and future studies need to include many more female patients. Nevertheless, our results showed that the immune system of women and men responds differently after neoadjuvant chemotherapy, and it needs to be considered if men and women should receive different therapy options, including sex-dependent immune issues [43].

### 4.4. Limitations of the Study

The comparison of our neoadjuvantly treated collective was not 1:1 randomized and 100% comparable with the chemotherapy naïve cohort [21,35], as the number of proximal tumors increased over time. Additionally, we have no data of CTLs in the treatment naïve cohort, limiting comparability. Regarding not having CTL data in the treatment naïve cohort, it was not possible to define the TIME for the former cohort. We only used FFPE tissue samples, considering the retrospective and observational character of our study, and we were unable to provide any functional data analysis. Unfortunately, in our study we only had 39 (22.5%) female patients. This gender imbalance might have compromised our data analysis. However, our morphological and statistical analyses could help to formulate novel hypotheses and inspire new experimental studies on tumor biological mechanisms operating in the tumor microenvironment after neoadjuvant/perioperative chemotherapy.

## 5. Conclusions

Hopefully, personalized medicine will become the mainstay for the treatment of GC. This should include the consideration of interindividual and gender specific differences of the TIME in GC, especially for the future development of adjuvant and palliative treatment with immune-checkpoint inhibitors.

## Figures and Tables

**Table 1:** Clinicopathological patient characteristics of the neoadjuvantly treated gastric cancer cohort and the treatment naïve cohort [21].

Clinicopathological Patient Characteristics		Neoadjuvantly Treated Gastric Cancer Cohort		Treatment Naive Gastric Cancer Cohort	
		**n**	**(%)**	**n**	**(%)**
**Study patients**		173		449	
**Age**	valid/missing	173	0	449	0
<64/<68 years		87	(50.3)	221	(49.2)
≥64/≥68 years		86	(49.7)	228	(50.8)
**Sex**	valid/missing	173	0	449	0
Men		134	(77.5)	285	(63.5)
Women		39	(22.5)	164	(36.5)
**Localization**	valid/missing	173	0	440	9
Proximal		120	(69.4)	143	(31.8)
Distal		53	(30.6)	297	(66.1)
**Laurén type**	valid/missing	173	0	449	0
Intestinal		79	(45.7)	239	(53.2)
Diffuse		30	(17.3)	136	(30.3)
Mixed		33	(19.1)	29	(6.5)
Unclassified		18	(10.4)	45	(10)
Complete remission		13	(7.5)	0	(0)
**T-category**	valid/missing	173	0	449	0
ypT0/pT0		17	(9.8)	0	(0)
ypT1/pT1		24	(13.9)	52	(11.6)
ypT2/pT2		23	(13.3)	53	(11.8)
ypT3/pT3		98	(56.6)	185	(41.2)
y/pT4/pT4		11	(6.4)	159	(35.4)
**N-category**	valid/missing	173	0	449	0
ypN0/pN0		62	(35.8)	126	(28.1)
ypN1/pN1		43	(24.9)	67	(14.9)
ypN2/pN2		42	(24.3)	78	(17.4)
ypN3/pN3		26	(15.0)	178	(39.6)
**M-category**	valid/missing	173	0	449	0
yM0/yM0		165	(95.4)	364	(81.1)
yM1/M1		8	(4.6)	85	(18.9)
**UICC stage (8th Edition)**	valid/missing	173	0	449	0
0/0/N+		13	(7.5)	0	(0)
IA/B		26	(15)	74	(16.5)
IIA/B		29	(16.8)	101	(22.5)
IIIA/B/C		88	(50.9)	189	(42.1)
IV		17	(9.8)	85	(18.9)
**L-category**	valid/missing	173	0	423	26
ypL0/pL0		123	(71.1)	202	(45)
ypL1/pL1		50	(28.9)	221	(49.2)
**V-category**	valid/missing	173	0	422	27
ypV0/pV0		162	(93.6)	374	(83.3)
ypV1/pV1		11	(6.4)	48	(10.7)
**R-category**	valid/missing	173	0	436	13
pR0		154	(89)	382	(85.1)
pR1/2		17	(9.8)	54	(12)
pRX		2	(1.2)	0	(0)
**Tumor regression grade (TRG)**	valid/missing	173	0		
TRG1a/b		56	(32.4)		
TRG2		28	(16.2)		
TRG3		89	(51.4)		

**Table 2:** Densities of tumor-associated neutrophils (TAN; naïve vs. neoadjuvant) and the densities of cytotoxic T cells (CTL) in five different compartments of gastric cancer. **The median is marked in bold.** The *p*-values denote the results of the Mann–Whitney test between the treatment naïve and the neoadjuvant cohort, as indicated by the superscribed prefix. The data for the treatment naïve cohort were retrieved from Clausen et al. [21]. CTL were not available from the treatment naïve cohort. The prefix symbols indicate which TAN data were analyzed statistically and to which the *p*-values correlated.

Histoanatomical Site	Density (n/mm²)	Treatment Naive Cohort TAN	Neoadjuvant Cohort TAN	Neoadjuvant Cohort CTL	*p*-Value
**Mucosa**	N	263 *	108 *	94	* *p* < 0.001
	25%-Percentile	25.1	54.9	144.4	
	Median	**57.6**	**132.7**	**298.1**	
	75%-Percentile	121.1	252.4	531.1	
	Range	2.0–2022.4	1.8–1495.9	9.6–1739.8	
**Tumor surface**	N	365 °	41 °	42	° *p* = 0.006
	25%-Percentile.	481.2	261.7	100.4	
	Median	**872.6**	**486.6**	**221.4**	
	75%-Percentile	1430.1	1159.4	636.7	
	Range	5.8–4127.0	63.5–3186.9	29.0–1855.5	
**Tumor center**	N	470 ^§^	157 ^§^	157	^§^*p* = 0.426
	25%-Percentile	47.4	63.2	119.7	
	Median	**130.1**	**109.5**	**296.2**	
	75%-Percentile	404.1	240.6	558.3	
	Range	3–5113.4	6.1–3336.7	6.0–1850.9	
**Invasion front**	N	390 ^#^	102 ^#^	93	^#^*p* = 0.003
	25%-Percentile	74.2	48.0	163.4	
	Median	**226.8**	**134.8**	**420.7**	
	75%-Percentile	723.6	414.5	826.9	
	Range	0–6711.0	2.5–2729.8	12.7–2644.7	
**Tumor scar**	N		54	53	
	25%-Percentile		18.7	34.9	
	Median		**36.8**	**79.5**	
	75%-Percentile		65.9	215.5	
	Range		4.7–314.4	4.6–561.8	

**Table 3:** Correlation between tumor immune microenvironment (TIME) and clinicopathological patient characteristics. (1) Fisher’s exact test. (2) Kendall’s tau test. * Significant after multiple testing in bold.

		Total		Infiltrated-Excluded		Infiltrated-Inflamed		TLS-TIME		*p*-Value
		**n**	**(%)**	**n**	**(%)**	**n**	**(%)**	**n**	**(%)**	
**Gender**	n	133		30	(22.6)	75	(56.4)	28	(21.1)	0.596 (1)
Female		28	(21.1)	6	(21.4)	18	(64.3)	4	(14.3)	
Male		105	(78.9)	24	(22.9)	57	(54.3)	24	(22.9)	
**Age**	n	133		30	(22.6)	75	(56.4)	28	(21.1)	0.197 (1)
≥64		68	(51.1)	17	(25.0)	41	(60.3)	10	(14.7)	
<64		65	(48.9)	13	(20.0)	34	(52.3)	18	(27.7)	
**Laurén Type**	n	133		30	(22.6)	75	(56.4)	28	(21.1)	0.127 (1)
Intestinal		70	(52.6)	15	(21.4)	42	(60.0)	13	(18.6)	
Diffuse		22	(16.5)	3	(13.6)	10	(45.5)	9	(40.9)	
Mixed		28	(21.1)	8	(26.6)	14	(50.0)	6	(21.4)	
Unclassified		13	(9.7)	4	(30.8)	9	(69.2)	0	(0.0)	
**ypT-category**	n	133		30	(22.6)	75	(56.4)	28	(21.1)	0.840 (1)
ypT0		2	(1.5)	0	(0.0)	2	(100.0)	0	(0.0)	
ypT1a/b		21	(15.8)	2	(9.5)	14	(66.7)	5	(23.8)	
ypT2		20	(15.0)	6	(30.0)	11	(55.0)	3	(15.0)	
ypT3		81	(60.9)	20	(24.7)	43	(53.1)	18	(22.2)	
ypT4a/b		9	(6.7)	2	(22.2)	5	(55.6)	2	(22.2)	
**ypN-category**	n	133		30	(22.6)	75	(56.4)	28	(21.1)	0.452 (1)
ypN0		41	(30.8)	7	(17.1)	22	(53.7)	12	(29.3)	
ypN1		37	(27.8)	11	(29.7)	18	(48.6)	8	(21.6)	
ypN2		35	(26.3)	7	(20.0)	24	(68.6)	4	(11.4)	
ypN3a/b		20	(15.0)	5	(25.0)	11	(55.0)	4	(20.0)	
**Localisation**	n	133		30	(22.6)	75	(56.4)	28	(21.1)	0.134 (1)
Proximal stomach		92	(69.1)	23	(25.0)	54	(58.7)	15	(16.3)	
Distal stomach		41	(30.8)	7	(17.1)	21	(51.2)	13	(31.7)	
**M-Stage**	n	133		30	(22.6)	75	(56.4)	28	(21.1)	1.000 (1)
M0		127	(95.4)	29	(22.8)	71	(55.9)	27	(21.3)	
M1		6	(4.6)	1	(16.7)	4	(66.7)	1	(16.7)	
**UICC Stage (8th Edition)**	n	133		30	(22.6)	75	(56.4)	28	(21.1)	0.516 (1)
IA/B		23	(17.3)	2	(8.7)	14	(60.9)	7	(30.4)	
IIA/B		26	(19.5)	6	(23.1)	13	(50.0)	7	(26.9)	
IIIA/B/C		70	(52.6)	18	(25.7)	40	(57.1)	12	(17.1)	
IV		14	(10.5)	4	(28.6)	8	(57.1)	2	(14.3)	
**ypL category**	n	133		30	(22.6)	75	(56.4)	28	(21.1)	0.022 (1)
ypL0		93	(69.9)	15	(16.1)	55	(59.1)	23	(24.7)	
ypL1		40	(30.1)	15	(37.5)	20	(50.0)	5	(12.5)	
**ypV category**	n	133		30	(22.6)	75	(56.4)	28	(21.1)	0.650 (1)
ypV0		124	(93.2)	27	(21.8)	71	(57.3)	26	(21.0)	
ypV1		9	(6.8)	3	(33.3)	4	(44.4)	2	(22.2)	
**Pn-Category**	n	133		30	(22.6)	75	(56.4)	28	(21.1)	1.000 (1)
Pn0		92	(69.1)	22	(22.4)	55	(56.1)	21	(21.4)	
Pn1		41	(30.8)	8	(22.9)	20	(57.1)	7	(20.0)	
**Resection**	n	133		30	(22.6)	75	(56.4)	28	(21.1)	0.730 (1)
R0		116	(87.2)	27	(23.3)	64	(55.2)	25	(21.6)	
R1		15	(11.2)	2	(13.3)	10	(66.7)	3	(20.0)	
RX		2	(1.5)	1	(50.0)	1	(50.0)	0	(0.0)	
**Tumor regression grade**	n	133		30	(22.6)	75	(56.4)	28	(21.1)	0.003 (1)
TRG1a/b		35	(26.3)	2	(5.7)	23	(65.7)	10	(28.6)	
TRG2		21	(15.8)	3	(14.3)	10	(47.6)	8	(38.1)	
TRG3		77	(57.9)	25	(32.5)	42	(54.5)	10	(13.0)	
**Mucosa CTL Density**	n	66		16	(24.6)	35	(53.0)	15	(22.7)	0.895 (1)
low		31	(47.0)	7	(22.6)	16	(51.6)	8	(25.8)	
high		35	(53.0)	9	(25.7)	19	(54.3)	7	(20.0)	
**Tumor Surface CTL Density**	n	36		8	(22.2)	21	(58.3)	7	(19.4)	0.097 (1)
low		16	(44.4)	1	(6.3)	12	(75.0)	3	(18.8)	
high		20	(55.6)	7	(35.0)	9	(45.0)	4	(20.0)	
**Tumor CTL Density**	n	132		30	(22.7)	74	(56.1)	28	(21.2)	0.004 (1)
low		55	(41.7)	9	(16.4)	40	(72.7)	6	(10.9)	
high		77	(58.3)	21	(27.3)	34	(44.2)	22	(28.6)	
**Invasion front CTL Density**	n	80		29	(36.2)	38	(47.5)	13	(16.3)	<0.001 (1) *
low		38	(47.5)	6	(15.8)	26	(68.4)	6	(15.8)	
high		42	(52.5)	23	(54.8)	12	(28.6)	7	(16.7)	
**Tumor scar CTL Density**	n	34		5	(14.7)	18	(52.9)	11	(32.4)	0.805 (1)
low		19	(55.9)	3	(15.8)	11	(57.9)	5	(26.3)	
high		15	(44.1)	2	(13.3)	7	(46.7)	6	(40.0)	

**Table 4:** Correlation of the densities of tumor-associated neutrophils and cytotoxic T cells divided into men and women, with tumor regression grade (TRG) according to Becker. (2) Kendall’s tau test.

Tumor-Associated Neutrophils		Total		Mucosa				Tumor Surface				Tumor Center				Invasion Front				Tumor Scar			
				**Q** **_1_**		**Q** **_2_** **_3_** **_4_**		**Q** **_1_**		**Q** **_2_** **_3_** **_4_**		**Q** **_1_**		**Q** **_2_** **_3_** **_4_**		**Q** **_1_**		**Q** **_2_** **_3_** **_4_**		**Q** **_1_**		**Q** **_2_** **_3_** **_4_**	
		n	(%)	n	(%)	n	(%)	n	(%)	n	(%)	n	(%)	n	(%)	n	(%)	n	(%)	n	(%)	n	(%)
**Male**	n *p*-Value (2)	134		83			0.069	31			0.637	123			0.008	84			0.154	41			0.641
TRG1a/1b		43	(32.1)	4	(13.3)	26	(86.7)	1	(16.7)	5	(83.3)	13	(40.6)	19	(59.4)	7	(43.8)	9	(56.3)	5	(22.7)	17	(77.3)
TRG2		19	(14.2)	4	(28.6)	10	(71.4)	2	(66.7)	1	(33.3)	6	(31.6)	13	(68.4)	2	(15.4)	11	(84.6)	3	(37.5)	5	(62.5)
TRG3		72	(53.7)	13	(33.3)	26	(66.7)	4	(18.2)	18	(81.8)	12	(16.7)	60	(83.3)	11	(20.0)	44	(80.0)	1	(9.1)	10	(90.9)
**Female**	n *p*-Value (2)	39		25			1.000	10			1.000	34			0.346	18			0.015	13			0.790
TRG1a/1b		13	(33.3)	3	(30.0)	7	(70.0)	0	(0.0)	0	(0.0)	3	(37.5)	5	(62.5)	2	(100.0)	0	(0.0)	1	(16.7)	5	(83.3)
TRG2		9	(23.1)	0	(0.0)	5	(100.0)	1	(33.3)	2	(66.7)	2	(22.2)	7	(77.8)	2	(40.0)	3	(60.0)	3	(60.0)	2	(40.0)
TRG3		17	(43.6)	3	(30.0)	7	(70.0)	2	(28.6)	5	(71.4)	3	(17.6)	14	(82.4)	1	(9.1)	10	(90.9)	0	(0.0)	2	(100.0)
**Cytotoxic T cells**		**Total**		**Mucosa**				**Tumor surface**				**Tumor center**				**Invasion front**				**Tumor scar**			
				**Q** **_1_** **_2_**		**Q** **_3_** **_4_**		**Q** **_1_** **_2_**		**Q** **_3_** **_4_**		**Q** **_1_** **_2_**		**Q** **_3_** **_4_**		**Q** **_1_** **_2_**		**Q** **_3_** **_4_**		**Q** **_1_** **_2_**		**Q** **_3_** **_4_**	
		n	(%)	n	(%)	n	(%)	n	(%)	n	(%)	n	(%)	n	(%)	n	(%)	n	(%)	n	(%)	n	(%)
**Male**	n *p*-Value (2)	134		71			0.576	32			0.895	123			0.871	77			0.005	39			0.151
TRG1a/b		43	(32.1)	16	(57.1)	12	(42.9)	4	(50.0)	4	(50.0)	18	(56.3)	14	(43.8)	12	(75.0)	4	(25.0)	9	(42.9)	12	(57.1)
TRG2		19	(14.2)	5	(41.7)	7	(58.3)	1	(33.3)	2	(66.7)	9	(47.4)	10	(52.6)	9	(75.0)	3	(25.0)	5	(62.5)	3	(37.5)
TRG3		72	(53.7)	15	(48.4)	16	(51.6)	9	(42.9)	12	(57.1)	38	(52.8)	34	(47.2)	20	(40.8)	29	(59.2)	7	(70.0)	3	(30.0)
**Female**	n *p*-Value (2)	39		23			0.674	10			1.000	34			0.596	16			0.005	14			1.000
TRG1a/b		13	(33.3)	6	(60.0)	4	(40.0)	0	(0.0)	1	(100.0)	3	(37.5)	5	(62.5)	2	(100.0)	0	(0.0)	3	(42.9)	4	(57.1)
TRG2		9	(23.1)	0	(0.0)	4	(100.0)	3	(100.0)	0	(0.0)	3	(33.3)	6	(66.7)	3	(75.0)	1	(25.0)	2	(40.0)	3	(60.0)
TRG3		17	(43.6)	5	(55.6)	4	(44.4)	4	(66.7)	2	(33.3)	8	(47.1)	9	(52.9)	1	(10.0)	9	(90.0)	1	(50.0)	1	(50.0)

## Data Availability

The data presented in this study are available on request from the corresponding author.

## Supplementary Materials

The following are available online at https://www.mdpi.com/article/10.3390/jpm11111184/s1, Table S1: Neoadjuvant/perioperative treatment (not specified denotes that the treatment protocol was not further specified in the records). Table S2: Correlation of the densities of tumor-associated neutrophils dichotomized into quartile 1 vs. quartiles 2–4 with clinicopathological patient characteristics. (1) Fisher’s exact test. (2) Kendall’s tau test. (3) Log-rank test. Table S3: Correlation of the densities of cytotoxic T cells dichotomized into quartiles 1 and 2 vs. quartiles 3 and 4 with clinicopathological patient characteristics. (1) Fisher’s exact test. (2) Kendall’s tau test. (3) Log-rank test.
