# Supplementary material for: Neoadjuvant/Perioperative Treatment Affects Spatial Distribution and Densities of Tumor Associated Neutrophils and CD8+ Lymphocytes in Gastric Cancer
Source: J Pers Med. 2021 Nov 12;11(11):1184. doi: 10.3390/jpm11111184 (PMC8622782; doi:10.3390/jpm11111184)
Supplement: Supplementary file 1 [file jpm-11-01184-s001.zip › jpm-1421136-supplementary.pdf]

Table S1: Neoadjuvant/perioperative treatment (not specified denotes that the treatment protocol was not further specified in the records)

TAN_Cohort_ID	Chemo neoadjuvant/perioperative pre-surgery	Chemo neoadjuvant/perioperative after surgery
AH001	not specified	not specified
AH002	not specified	not specified
AH003	Cisplatin / 5-FU / Paclitaxel 3 cycles	0
AH004	Carboplatin/5-FU, 3 cycles	Vinorelbin 7 cycles
AH005	not specified	not specified
AH006	not specified	not specified
AH007	not specified	not specified
AH008	not specified	not specified
AH009	EOX 2 cycles	EOX 1 cycle; therapy stopped due to side effects
AH010	neoadjuvant radiochemo	not specified
AH011	ECX 3 cycles	ECX 2 cycles
AH012	EOX 3 cycles	0
AH013	EOX 3 cycles	EOX 1 cycle
AH014	EOX 3 cycles	EOX 3 cycles
AH015	EOX 3 cycles	EOX 2 cycles
AH016	not specified	not specified
AH017	not specified	not specified
AH018	EOX 3 cycles	0
AH019	DCX 3 cycles	FOLFOX6 2 cycles
AH020	DCX-AOI-study 3 cycles	recommended, lost to follow-up
AH021	not specified	not specified
AH022	DCX-AIO study 3 cycles	0
AH023	not specified	6 cycles, not further specified
AH024	Carboplatin + Docetaxel + Capecitabine 3 cycles	Carboplatin + Docetaxel 2 cycles
AH025	not specified	not specified
AH026	DCX-AOI-study 3 cycles	0
AH027	FLOT 5 cycles	FLOT 1 cycle
AH028	FOLFOX 6 cycles	0
AH029	not specified	not specified
AH030	not specified	not specified
AH031	FLOT 4 cycles	0
AH032	FLOT 4 cycles	0
AH033	FLO 4 cycles	3 x FLO, 1 xFLOT
AH034	FLOT 4 cycles	FLOT 4 cycles
AH035	FLOT 1 cycle	0
AH036	FLOT 4 cycles	0
AH037	EOX 3 cycles	EOX 3 cycles
AH038	FLOT 7 cycles	recommended, lost to follow-up
AH039	not specified	0
AH040	ECX ? cycles	0
AH041	FLOT 4 cycles	not specified
AH042	FLOT 4 cycles	0
AH043	not specified	not specified
AH044	not specified	0
AH045	FLOT 4 cycles	0
AH046	FLOT 4 cycles	0
AH047	FLOT 4 cycles	0
AH048	EOX 3 cycles	0
AH049	ECF 3 cycles	ECF, number of cycles unknown
AH050	FLOT 4 cycles	0
AH051	EOX 3 cycles	0
AH052	not specified	0
AH053	not specified	not specified
AH054	not specified	not specified
AH055	not specified	not specified
AH056	not specified	not specified
AH057	not specified	not specified
AH058	not specified	0
AH059	EOX 3 cycles	EOX 3 cycles
AH060	EOX 3 cycles	EOX 3 cycles
AH061	FLO 3 cycles	0
AH062	EOX 3 cycles	not specified
AH063	EOX 3 cycles	0
AH064	ECF 1 cycle, FLO 3 cycles	0
AH065	FLOT 4 cycles	FLOT 2 cycles (discontinuation due to side effects)
AH066	not specified	0

AH067	not specified	0
AH068	not specified	not specified
AH069	FLOT 4 cycles	0
AH070	FLOT 3 cycles	0
AH071	FLO 3 cycles	0
AH072	not specified	not specified
AH073	not specified	not specified
AH074	EOX 3 cycles	not specified
AH075	FLOT 4 cycles	0
AH076	FLO 4 cycles	0
AH077	Carboplatin + Paclitaxel + 41 Gy	0
AH078	FLOT 4 cycles	FLO 3 cycles
AH079	FLO 3 cycles	0
AH080	FLOT 8 cycles	0
AH081	CROSS study (41,4 Gy; 5x Carboplatin+Paclitaxel)	0
AH082	FLOT 4 cycles	Radiochemo (54 Gy, Carboplatin/Vinorelbin)
AH083	FLOT 5 cycles	FLOT 3 cycles
AH084	FLOT 4 cycles	0
AH085	CROSS study (41,4 Gy; 5x Carboplatin+Paclitaxel)	0
AH086	CROSS study (41,2 Gy; 5x Carboplatin+Paclitaxel)	0
AH087	Cisplatin/ 5-FU 10 cycles	0
AH088	not specified	0
AH089	FLOT 4 cycles	FLOT 4 cycles
AH090	FLOT 3 cycles	FLOT 4 cycles
AH091	EOX 3 cycles	EOX (cycleszahl nicht dokumentiert)
AH092	EOX 3 cycles	0
AH093	FLOT 4 cycles	0
AH094	FLOT 4 cycles	FLO 4 cycles
AH095	FLOT 4 cycles	0
AH096	FLOT 4 cycles	EOX 3 cycles
AH097	FLOT + Herceptin 4 cycles	FLOT 4 cycles
AH098	FLOT 4 cycles	0
AH099	FLOT 4 cycles	FLOT 4 cycles
AH100	FLOT 4 cycles	FLOT 1 cycle
AH101	FLOT 4 cycles	FLOT 4 cycles
AH102	not specified	not specified
AH103	FLOT 4 cycles o. FLO?	0
AH104	FLOT 4 cycles	FLOT 4 cycles
AH105	not specified	not specified
AH106	FLOT 4 cycles	0
AH107	FLOT 4 cycles	0
AH108	Radiochemo	0
AH109	FLOT 1 cycle	Radiochemo (50,4Gy total dosis, Carboplatin / Paclitaxel)
AH110	RCTX CROSS (Carboplatin/Paclitaxel)	not specified
AH111	not specified	not specified
AH112	FLOT 4 cycles	0
AH113	FLOT 4 cycles	0
AH114	not specified - 3 cycles	recommended, lost to follow-up
AH115	not specified	0
AH116	FLOT 4 cycles	ARTIST study: radiochemo with Capecitabine + Cisplatin
AH117	FLOT (number of cycles unknown)	0
AH118	FLO 4 cycles	0
AH119	FLO 4 cycles (dosis reduced due impaired renal function)	0
AH120	FLOT 4 cycles	recommended, lost to follow-up
AH121	EOX (number of cycles unknown)	0
AH122	EOX	0
AH123	1 cycle of unkknown scheme	0
AH124	FLOT 4 cycles	0
AH125	ECF 3 cycles	ECF 3 cycles
AH126	FLOT+Herceptin (1 cycle as part of Herceptin-FLOT-study, subsequently surgery since progress)	Radiochemo (50Gy total dosis, Carboplatin/Paclitaxel)
AH127	FLOT 3 cycles	
AH128	neoadj. Radiochemo with Taxol/Carboplatin	0
AH129	FLOT 4 cycles	FLOT 3 cycles
AH130	EOX 3 cycles	0
AH131	Started with FLOT and discontinuation under progress	0
AH132	EOX 3 cycles	0
AH133	FLOT 3 cycles	palliative Chemo due to metastases

AH134	FLOT (4 cycles)	FLO (no Docetaxel due to age)
AH135	FLOT	FLOT 4 cycles + Rx 50.4 Gy
AH136	FLOT 4 cycles	FLOT 4 cycles
AH137	FLOT 4 cycles	FLOT 2 cycles (Keine Fortsetzung auf Pat. Wunsch)
AH138	RCTX: ESOPEC study (arm B; 5*1,8 Gy; cumul. 41.4 Gy, Carboplatin AUC2, Paclitaxel 50mg/m2 in total 5* parallel)	0
AH139	Oxaliplatin /5FU	recommended, lost to follow-up
AH140	FLOT 4 cycles	0
AH141	FLOT 4 cycles	0
AH142	FLOT 4 cycles	
AH143	FLOT 4 cycles	
AH144	CROSS-study	
AH145	FLOT 4 cycles	
AH146	CROSS-study	
AH147	not specified	
AH148	FLOT 4 cycles	
AH149	RTCX with Taxol/Carboplatin, radiation with 45.0 Gy total dosis 1.8 Gy single dose	
AH150	CROSS-study	
AH151	CROSS-study	
AH152	FLOT 4 cycles	
AH153	FLOT 4 2 cycles	
AH154	CROSS-study	
AH155	Paclitaxel/Carboplatin 50/AUC2, radiation with 1,8 Gy single dose 5x/week; 41.4 Gy total radiation	
AH156	FLOT 4 cycles	
AH157	FLO 5 cycles	
AH158	0	
AH159	0	
AH160	0	
AH161	FLOT 3 cycles	
AH162	FLOT 4 cycles	
AH163	FLOT 4 cycles	
AH164	(Her-)FLOT (number of cycles not documented)	
AH165	FLOT	
AH166	FLOT 4 cycles	
AH167	FLOT 4 cycles	
AH168	FLOT 4 cycles (1. cycle port thrombosis; 2. cycle without 5-FU, cycle 3 +4 with Xelox)	
AH169	FLOT 4 cycles + Herceptin	
AH170	FLOT 4 cycles	
AH171	FLOT 4 cycles	
AH172	FLOT 4 cycles + Herceptin	
AH173	FLOT 4 cycles	
